# Improved Prognosis and Low Failure Rate with Anticoagulation as First-Line Therapy in Mesenteric Venous Thrombosis

**DOI:** 10.1007/s00268-018-4667-x

**Published:** 2018-05-17

**Authors:** S. Salim, M. Zarrouk, J. Elf, A. Gottsäter, O. Ekberg, S. Acosta

**Affiliations:** 1 Department of Clinical Sciences, Lund University, Malmö, Sweden; 2 Department of Translational Medicine, Division of Medical Radiology, Lund University, Malmö, Sweden; 3 Vascular Centre, Department of Cardio-Thoracic and Vascular Surgery, Skåne University Hospital, 205 02 Malmö, Sweden

## Abstract

**Background:**

Monotherapy with anticoagulation has been considered as first-line therapy in patients with mesenteric venous thrombosis (MVT). The aim of this study was to evaluate outcome, prognostic factors, and failure rate of anticoagulation as monotherapy, and to identify when bowel resection was needed.

**Methods:**

Retrospective study of consecutive patients with MVT diagnosed between 2000 and 2015.

**Results:**

The overall incidence rate of MVT was 1.3/100,000 person-years. Among 120 patients, seven died due to autopsy-verified MVT without bowel resection and 15 underwent immediate bowel resection without prior anticoagulation therapy. The remaining 98 patients received anticoagulation monotherapy, whereof 83 (85%) were treated successfully. Fifteen patients failed on anticoagulation monotherapy, of whom seven underwent bowel resection and eight endovascular therapy. Endovascular therapy was followed by bowel resection in three patients. Two late bowel resections were performed due to intestinal stricture. The 30-day mortality rate was 19.0% in the former (2000–2007) and 3.2% in the latter (2008–2015) part of the study period (*p *= 0.006). Age ≥75 years (OR 12.4, 95% CI [2.5–60.3]), management during the former as opposed to the latter time period (OR 8.4, 95% CI [1.3–54.7]), and renal insufficiency at admission (OR 8.0, 95% CI [1.2–51.6]) were independently associated with increased mortality in multivariable analysis.

**Conclusions:**

Short-term prognosis in patients with MVT has improved. Contemporary data show that monotherapy with anticoagulation is an effective first choice in MVT patients.

## Background

Within the spectrum of patients presenting with acute abdominal pain, it is difficult to delineate those with mesenteric venous thrombosis (MVT). MVT is a rare but life-threatening condition which without treatment can develop into bowel ischemia, bowel gangrene, peritonitis, and death [1]. Main causes of MVT are coagulation disorders, abdominal inflammatory conditions, malignancies, and liver diseases [2]. Currently available investigations fail to identify a causal factor in about 20% of patients [3, 4]. Improved diagnostic workup with computed tomography (CT) may possibly lead to increased detection rates and earlier diagnosis of MVT [3, 4]. Immediate anticoagulation therapy after diagnosis has been proposed as the first-line treatment option [5].

To prospective study, MVT is challenging due to the low incidence of the condition, and large cohort studies would provide a valuable insight into the optimal management. This retrospective study was designed to evaluate prognostic factors and trends in prognosis over time in a large cohort of patients with MVT. Secondary aims were to evaluate the failure rate with anticoagulation as monotherapy, to identify when failures occurred, and when bowel resection was needed.

## Methods

Identification of all patients with MVT treated surgically or non-surgically in Malmö University Hospital between 2000 and 2015 based on the International Statistical Classification of Diseases and Related Health Problems (ICD), tenth edition, codes I81 (portal vein thrombosis [PVT] or MVT) and K55 (mesenteric ischemia), and *AuriculA* [6] (national quality register for anticoagulant treatment). Patient records and all CT images in patients with PVT or MVT as well as unclear cases of mesenteric ischemia were scrutinized. Patients with thrombosis in the superior mesenteric vein with or without anatomical involvement of portal or splenic veins were included in the present study. Patients diagnosed 2000–2006 have been reported upon previously [7]. The patient series was pragmatically divided at the study protocol stage into two periods, the former (2000–2007) and the latter (2008–2015), for analysis of changes in patient characteristics, risk factor profile, mode of diagnosis, and outcome. In emergencies, single-detector row CT was performed between 2000 and 2003, and multi-detector row CT from 2004 and onwards [8]. Mortality data were obtained from the Swedish Population Registry. Median follow-up after diagnosis for patients with MVT was 62 (interquartile range [IQR] 24–128) months. End of follow-up was September 29, 2017. The study was approved by the Research Ethical Review Board in Lund (Dnr 2015/143).

## Treatment strategy

After diagnosis of MVT with CT, the mainstay of treatment was conservative with immediate full anticoagulation with either intravenous heparin infusion or subcutaneous LMWH, full bowel rest, total parenteral nutrition, and analgesia. Patients admitted with peritonitis or rapid progression toward peritonitis underwent laparotomy and bowel resection. Patients not responding to anticoagulation underwent endovascular measures with or without local thrombolysis, and those not responding to this therapy was subjected to laparotomy. Clearly necrotic and demarcated bowels were resected and anastomosed. Bowels with unclear viability were usually evaluated at a second-look laparotomy, and bowel resections were followed by anastomoses or diverting stomas. Patients with identified transient risk factors were usually treated with oral anticoagulation for 6 months, whereas those with permanent risk factors or unidentified risk factors were prescribed lifelong anticoagulation. Up to 2014, the vitamin K antagonist (warfarin) was the only oral anticoagulation therapy, whereas direct-acting oral anticoagulants were gradually introduced as a treatment option from 2012.

### Definitions

Primary MVT is defined as an idiopathic condition, whereas secondary MVT is defined by an identified etiologic factor. Patients with abdominal pain of less than 4-week duration were classified as having acute MVT. Those with symptoms for more than 4 weeks, but without bowel infarction, and those with asymptomatic MVT diagnosed incidentally on abdominal imaging as clinically nonsignificant findings, were defined as chronic MVT. The term thrombophilia was used as a common denominator for factors that may promote MVT, such as coagulation disorders, malignancy, previous or concomitant venous thromboembolism, and use of oral anticonceptives or estrogen substitution. The presence of inherited thrombophilia such as Factor V Leiden mutation and acquired thrombophilia as JAK2 V617F (janus-activated kinase gain of function substitute of valine to phenylalanine at position 617) mutation was registered. Previous cardiovascular disease was defined as previous myocardial infarction, angina pectoris, history of coronary artery bypass grafting, percutaneous coronary intervention, stroke, or transient ischemic attack. Renal insufficiency was defined as a serum creatinine level higher than 105 μmol/l (1.2 mg/dl) in men and 90 μmol/l (1.0 mg/dl) in women.

### Statistical analysis

Data management and statistical analysis were performed using SPSS for Windows (SPSS, version 23.0, Chicago, Illinois, USA). Age and gender-specific total incidence rates were based on the number of patients diagnosed with MVT residing in Malmö, and expressed as number of cases per 100,000 person-years. Population data, overall and gender-specific, for Malmö in 2008 obtained from Statistics Sweden were used for calculation of incidence. Differences in proportions were evaluated using *χ*^2^ or Fisher’s exact test. Age was expressed as median (range). Variables associated with 30-day mortality (*p *< 0.1) were further tested in a multivariable binary logistic regression model and expressed in terms of odds ratios (OR) with 95% confidence interval (CI). *p* < 0.05 was considered significant.

## Results

### Incidence

One hundred and twenty patients, 67 men and 53 women, were diagnosed with MVT from 2000 to 2015. The overall incidence rate of MVT in Malmö was estimated to 1.3/100,000 person-years (1.4/100,000 person-years in men and 1.2/100,000 person-years in women).

### Patient characteristics

Median age at admission was 58 (range 19–95) years. Median body mass index (BMI) was 27.5 (IQR 25.2–30.0; *n *= 50) in men and 25.8 (IQR 23.7–33.4; *n *= 38) in women. Acute MVT was found in 115 (96%) patients, and primary and secondary MVT in 26 (22%) and 94 (78%) patients, respectively. Risk factors such as any direct injury to the vein due to disease or surgery were found in 35(29%) patients, local or systemic venous congestion in 19 (16%), and thrombophilia in 72(60%). Twenty (17%) patients had abdominal malignancies. History of previous venous thromboembolism was documented in 24 (20%) patients. Among 89 tested, 39 (44%) patients had positive tests for inherited or acquired coagulation disorder. The most common thrombophilia was activated protein C resistance (Factor V Leiden mutation), occurring in 22 (18%) patients (19 in heterozygous and three in homozygous genotype). In nine patients with myeloproliferative disease, eight (89%) were JAK-2 V617 mutation positive.

Patients diagnosed in the former period (2000–2007) were older (*p *= 0.013) and had higher proportions of abdominal malignancy (*p *= 0.009) and activated protein C resistance (*p *= 0.002) compared to those diagnosed in the latter period (2008–2015) (Table 1).

**Table 1:** Patient characteristics and risk factors for mesenteric venous thrombosis in the former (2000–2007) and the latter (2008–2015) parts of the study

Factors	Former period (*n *= 58)	Latter period (*n *= 62)	Univariable analysis (*p* value)
Median age (years; IQR)	64 (50–73)	54 (47–65)	0.013
Women (%)	27 (47)	42 (42)	0.61
Acute pancreatitis (%)	10 (17)	7 (11)	0.35
Recent abdominal surgery	5 (9)	3 (5)	0.35
*Thrombophilia*	40 (69)	32 (52)	0.053
History of previous venous thromboembolism	12 (21)	12 (19)	0.86
Abdominal malignancy	15 (26)	5 (8)	0.009
Positive test for inherited or acquired coagulation disorder	20/36 (56)	19/53 (36)	0.066
Activated protein C resistance (Factor V Leiden mutation)	15/36 (42)	7/53 (13)	0.002

### Mode of establishing diagnosis

During the latter time period, all patients were diagnosed by radiological imaging, in 97% of cases by CT with intravenous contrast enhancement. During this period, CT was more frequently used for MVT diagnosis compared to the former time period (*p *< 0.001) (Table 2). During the former time period, there were six autopsy-verified deaths in patients not undergoing bowel resection, of whom two died outside of hospital.

**Table 2:** Mode of establishing diagnosis in the former (2000–2007) and the latter (2008–2015) parts of the study

Factors	Former period (*n *= 58)	Latter period (*n *= 62)	Univariable analysis (*p* value)
Autopsy frequency (%)	25	12	<0.0001
*Primary mode of diagnosis*			
Autopsy	6 (10.3)	0 (0.0)	
Computed tomography (with intravenous contrast)	41 (70.7)	60 (96.8)	
Ultrasound	0 (0.0)	2 (3.2)	
Operation	11 (19.0)	0 (0.0)	<0.001
Bowel resection rate (excluding autopsy cases)	14/52 (26.9)	10 (16.1)	0.16

### Bowel resection and endovascular therapy

**Fig. 1:**
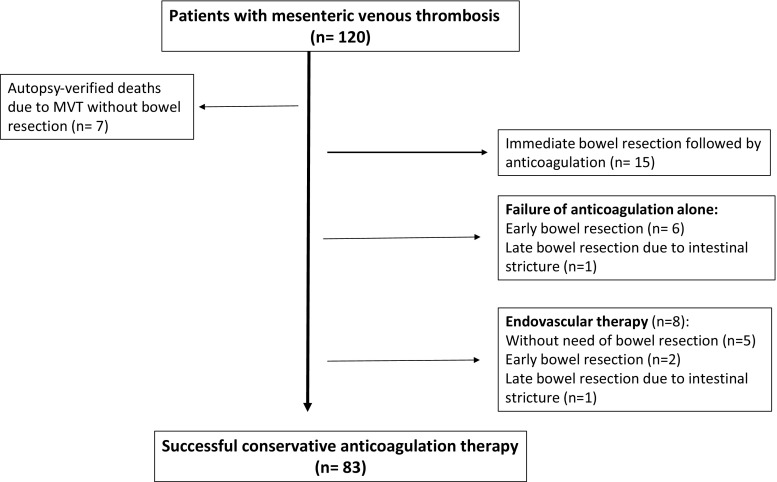
Management in patients with mesenteric venous thrombosis

**Fig. 2:**
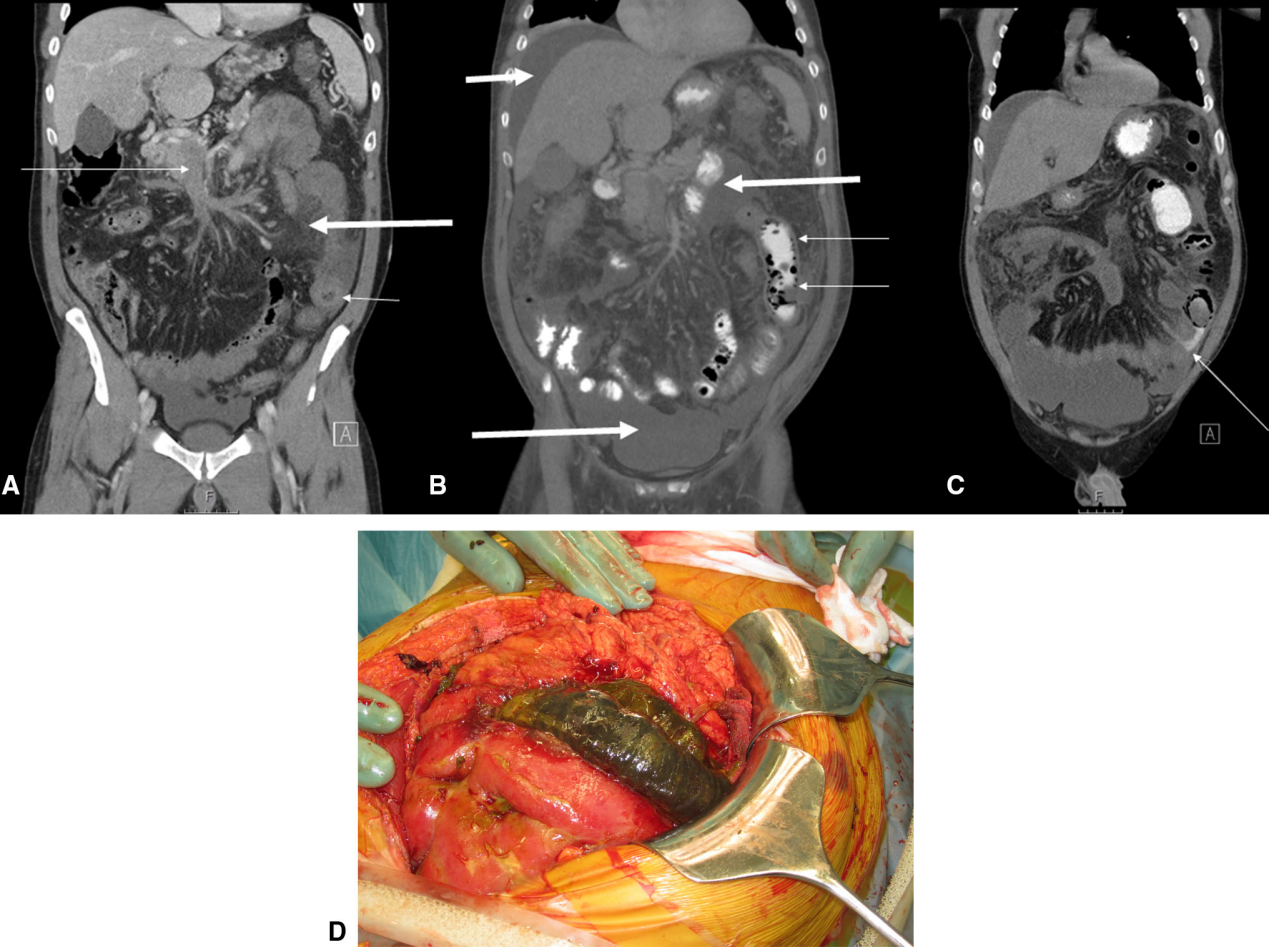
Failure of anticoagulation therapy. A 50-year-old male patient with a history of ulcerative proctitis who was admitted with 3 days of abdominal pain and C-reactive protein (CRP) of 161 mg/L. Diagnosis of mesenteric venous thrombosis (Fig. 2a, long thin arrow) was achieved after computed tomography (CT) with intravenous contrast enhancement and imaging in the portal/parenchymal phase. Note thickening of the jejunum (short arrow) and the mesenteric edema (long thick arrow). The patient had localized signs of peritonitis to the left in the abdomen and absent bowel sounds at the time of diagnosis. Full-dose heparin infusion was started, whereafter the patient improved temporarily but later deteriorated. A new CT (Fig. 2b) after 13 days of heparin therapy showed progression of ascites (thick arrows) and occurrence of gas bubbles (thin arrows) in the jejunal wall. Continued conservative therapy resulted in further clinical deterioration, and after 20 days of heparin therapy a CT (Fig. 2c) showed leakage of perorally administered contrast outside of the bowels (arrow). Explorative laparotomy showed a well-demarcated 1-meter-long transmural green necrosis of the jejunum (Fig. 2d) with a large perforation. The patient recovered after bowel resection, open abdomen therapy, and reanastomosis of the stapled bowel ends. Testing for thrombophilia showed that the patient was positive for JAK2 V617F mutation and a bone marrow biopsy diagnosed a polycythemia vera. The patient is scheduled for lifelong vitamin K antagonist therapy, and cytoreductive therapy with interferon, and is also undergoing regular venesection

**Fig. 3:**
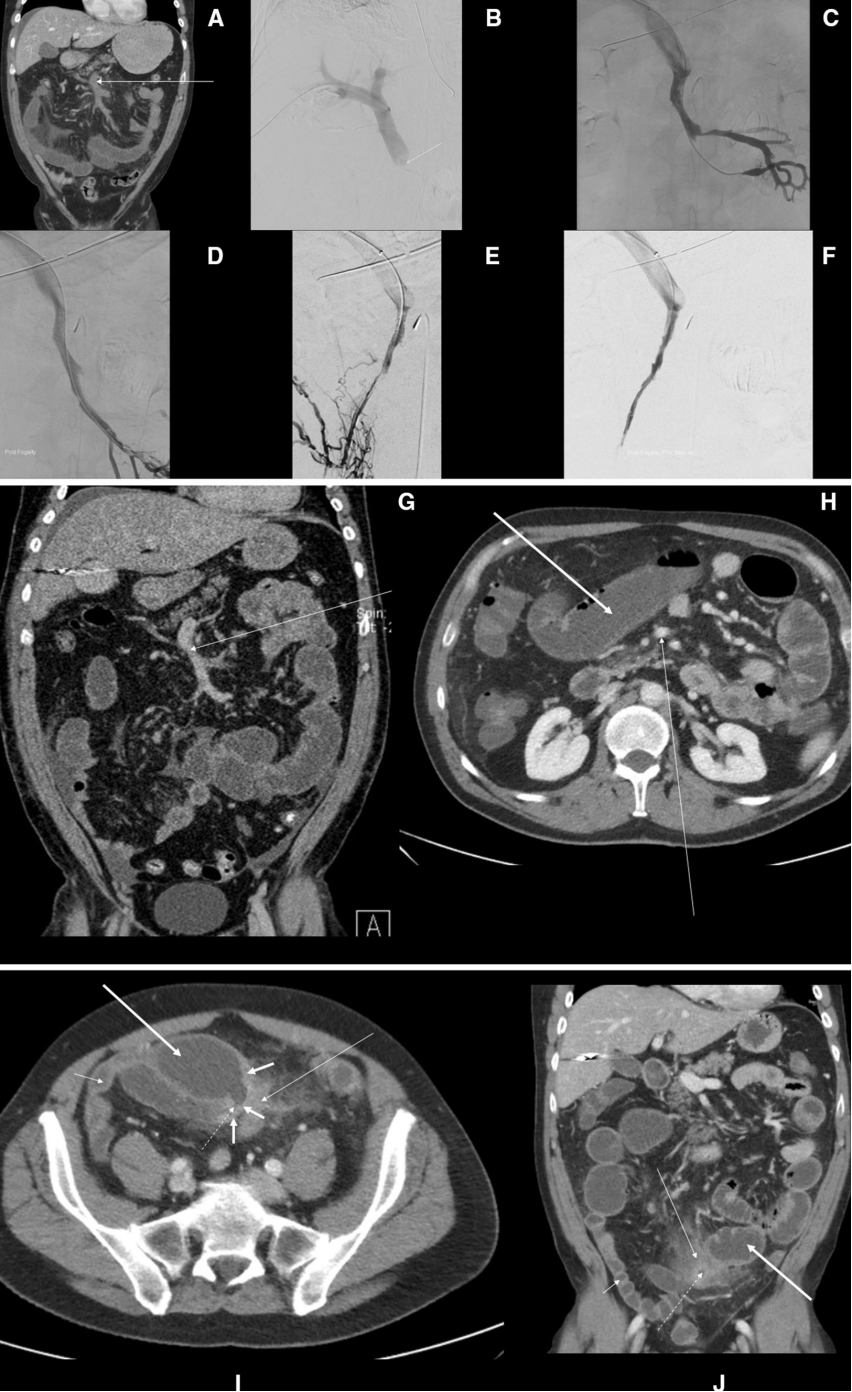
Endovascular therapy of mesenteric venous thrombosis after failure of anticoagulation treatment. A 53-year-old man with history of 3 months of anticoagulation treatment for deep venous thrombosis in the lower leg and Factor V Leiden mutation in the homozygous form. The patient fell ill with acute abdominal pain, and CT diagnosed an extensive MVT (**a**, arrow). He underwent transhepatic puncture and access to the portal vein. Venography showed total occlusion of the superior mesenteric vein (SMV) (**b**, arrow). Mechanical thrombectomy with an AngioJet® device (MEDRAD, Warrendale, Pennsylvania, USA) and endovascular Fogarty catheter thrombectomy were carried out, followed by thrombolysis with recombinant tissue plasminogen activator (rtPA) into the branches of the SMV and superior mesenteric artery. After a total dose of 25 mg rtPA over 25 h, improved flow in the SMV was noted. Endovascular rethrombectomy with a Fogarty catheter was performed owing to residual clots in the SMV branches (**c**–**f**). CT venography before discharge showed no signs of thrombus within the SMV and the proximal parts of the major venous branches (**g**, arrow). The patient did not recover fully and was readmitted after 3 months with symptoms of bowel obstruction. CT venography showed fully patent SMV (**h**, arrow), but severe localized fibrosis in the small bowel wall (**i**, thick short arrows) and adjacent mesenteric fat (**i**, **j**, thin long arrow) causing a bowel stricture. Note the narrow bowel lumen at the stricture (**i**, **j**, interrupted line). There is a prestenotic bowel dilatation (**h**, **i**, **j**, thick long arrow) and a poststenotic normalized bowel (**i**, **j**, thin short arrow). The patient underwent immediate bowel resection of the stricture, recovered and is on lifelong vitamin K antagonist medication

Bowel resection rates did not differ between the two periods (Table 2). Among the 98 patients receiving anticoagulation treatment, 83 (85%) were successfully treated with heparin as monotherapy without need for surgical intervention (Fig. 1). Throughout the study period, fifteen patients underwent explorative laparotomy and bowel resection without preoperative diagnosis, and another 15 patients underwent bowel resection (Fig. 2) or endovascular therapy due to failure of anticoagulation as monotherapy. Endovascular therapy was performed in eight patients, out of whom three underwent bowel resection. The two late bowel resections due to intestinal stricture were performed after 3 and 5 months, respectively, after index admission. The endovascular procedures performed were thrombolysis via the superior mesenteric artery (*n *= 4), transjugular intrahepatic portal shunt (TIPS) with stenting (*n *= 2), transjugular mechanical thrombectomy (AngioJet® device [MEDRAD, Warrendale, Pennsylvania, USA]) and thrombolysis (*n *= 1), transhepatic stenting (*n *= 1), transhepatic mechanical thrombectomy (AngioJet®), and Fogarty catheter balloon thrombectomy (*n *= 1). Another two TIPS procedures failed. Local thrombolysis via the superior mesenteric artery was not considered first option, but was used in combination with other endovascular therapies in three patients. In the fourth patient, TIPS was not considered an option due to the advanced extent of portomesenteric venous thrombosis, and 56 mg recombinant tissue plasminogen activator (rtPA) was continuously infused into the superior mesenteric artery over 60 h with success and without need of bowel resection. The sum of procedures performed exceeds eight, reflecting that a combination of techniques was often used. The median dose of thrombolytic agent, alteplase (Actilyse®; Boehringer, Ingelheim, Germany), administered locally in the mesenteric circulation, was 30 mg (range 14–56) in the four treated patients. One patient underwent a failed TIPS combined with thrombolysis via the superior mesenteric artery, complicated by perihepatic hematoma requiring explorative laparotomy for control of bleeding. Bowel resection due to late intestinal stricture was performed in two patients (Fig. 3). Lifelong anticoagulation therapy after successful non-operative management was given to 49% (17/35) of patients in the former period and 71% (34/48) in the latter (*p *= 0.040).

### Factors associated with 30-day mortality

Overall 30-day mortality rate was 10.8, 19.0% in the former time period versus 3.2% in the latter time period (*p *= 0.006). The 30-day mortality after surgery (bowel resection and/or endovascular therapy) was 12.5% (2/16) in the former period versus 7.1% (1/14) in the latter (*p *= 1.0). Age ≥75 years, management during the former as opposed to the later time period, pancreatic malignancy, and renal insufficiency at admission were all associated with increased 30-day mortality in univariable analysis. Age ≥75 years (OR 12.4, 95% CI [2.5–60.3]), management during the former time period as opposed to the latter period (OR 8.4, 95% CI [1.3–54.7]), and renal insufficiency at admission (OR 8.0, 95% CI [1.2–51.6]) were independently associated with increased mortality in the multivariable analysis (Table 3).

**Table 3:** Factors associated with 30-day mortality in 120 patients with mesenteric venous thrombosis

Factors	Number of patients	30-day mortality (%)	Univariable analysis (*p* value)	Multivariable analysis	
				OR (95% CI)	*p* value
All patients	120	10.8	–		
≥75 years	17	47.1	<0.001^a^	12.4 (2.5–60.3)	0.002
Female gender	53	17.0	0.054^a^	2.4 (0.5–11.7)	0.29
Period (2000–2007 vs. 2008–2015)	58 vs. 62	19.0 vs. 3.2	0.006^a^	8.4 (1.3–54.7)	0.026
Malignancy	23	17.4	0.26	–	
Abdominal malignancy	20	20.0	0.15	–	
Pancreatic malignancy	7	42.9	0.027^a^	5.1 (0.6–43.6)	0.13
Metastatic malignancy	14	28.6	0.045	–	
History of previous venous thromboembolism	24	12.5	0.77	–	
Activated protein C resistance	22/89	0.0	1.0	–	
Pancreatitis	17	0.0	0.21	–	
Liver cirrhosis	6	33.3	0.13	–	
Inflammatory bowel disease	7	0.0	1.0	–	
Renal insufficiency at admission	20	25	0.035^a^	8.0 (1.2–51.6)	0.029
Bowel resection	24	8.3	1.0	–	

^a^Entered into a multivariable logistic regression model

## Discussion

The adjusted results of the present population-based study on 120 patients showed that prognosis for patients with MVT improved during the study period. The increased diagnostic and therapeutic activity, including possibility to perform endovascular therapy, should be related to the current low 30-day mortality rate of 3.2%. Interestingly, the proportions of patients with activated protein C resistance [9], abdominal malignancy [10], and age were lower in the latter period, perhaps reflecting an increased activity in preventing venous thromboembolism including prophylactic anticoagulation therapy in high-risk patients. Since randomized trials comparing safety and efficacy of various treatments most likely will be impossible to conduct in these patients, evidence will rely upon prospective cohort studies. International, multicenter collaboration is necessary, as exemplified by the prospective study promoted by the International Society on Thrombosis and Hemostasis (ISTH), in which affiliated centers worldwide were invited to participate [11]. In similar future studies, in which a larger proportion of patients will likely receive endovascular therapy [12], it would be preferable to not only report on therapy-related major bleeding complications, thrombotic events, bowel necrosis, and mortality. High-quality data on patency rates of the portomesenteric venous system, and patient-reported outcomes such as quality of life and pain scores before and after conservative and endovascular therapy, would also be helpful to supply physicians and patients with important data to support decision making.

The overall incidence rate of MVT in Malmö was estimated to 1.3 per 100,000 person-years, a figure in the lower range of incidence reported in the 1970s [1]. This might partly be related to the markedly reduced autopsy frequency [13], from 85% [14] to 12% in the latter time period of the present study. On the other hand, important improvements in diagnostics and treatment of hypercoagulable states have occurred during this period [15] probably resulting in a decrease in venous thromboembolism. However, since MVT is very rarely suspected already in the emergency setting [16], or sometimes confused with arterial mesenteric ischemia at laparotomy, and with the contemporary low autopsy frequency in the population the contemporary true incidence is hard to estimate.

The decrease in 30-day mortality from 19.0% during the former half of the study period to 3.2% during the latter has several explanations. Earlier diagnosis by the use of available high-resolution, high-speed CT scanners around the clock in patients with unexplained abdominal pain would probably help to avoid development of bowel gangrene and peritonitis and the poor prognosis in these cases. CT with intravenous contrast and imaging in the portal phase is clearly the most accurate method of diagnosing the condition [17]. Corroborating other reports [18, 19], the present study showed that a non-operative approach with immediate anticoagulation therapy with unfractionated or low molecular weight heparin at the time of diagnosis was an effective treatment for acute MVT. Explorative laparotomy and bowel resection due to bowel gangrene and peritonitis will always be a way to rescue these patients in cases of rapid development of intestinal infarction, overlooked diagnosis, or late presentation as shown in Fig. 1. The clinician should also remember that the possibility of intestinal infarction is not ruled out until full resolution of pain occurs [2]. The study identified failure of anticoagulation therapy in a small proportion of patients, occurring mainly after days to weeks of medical therapy (Fig. 1).

**Fig. 4:**
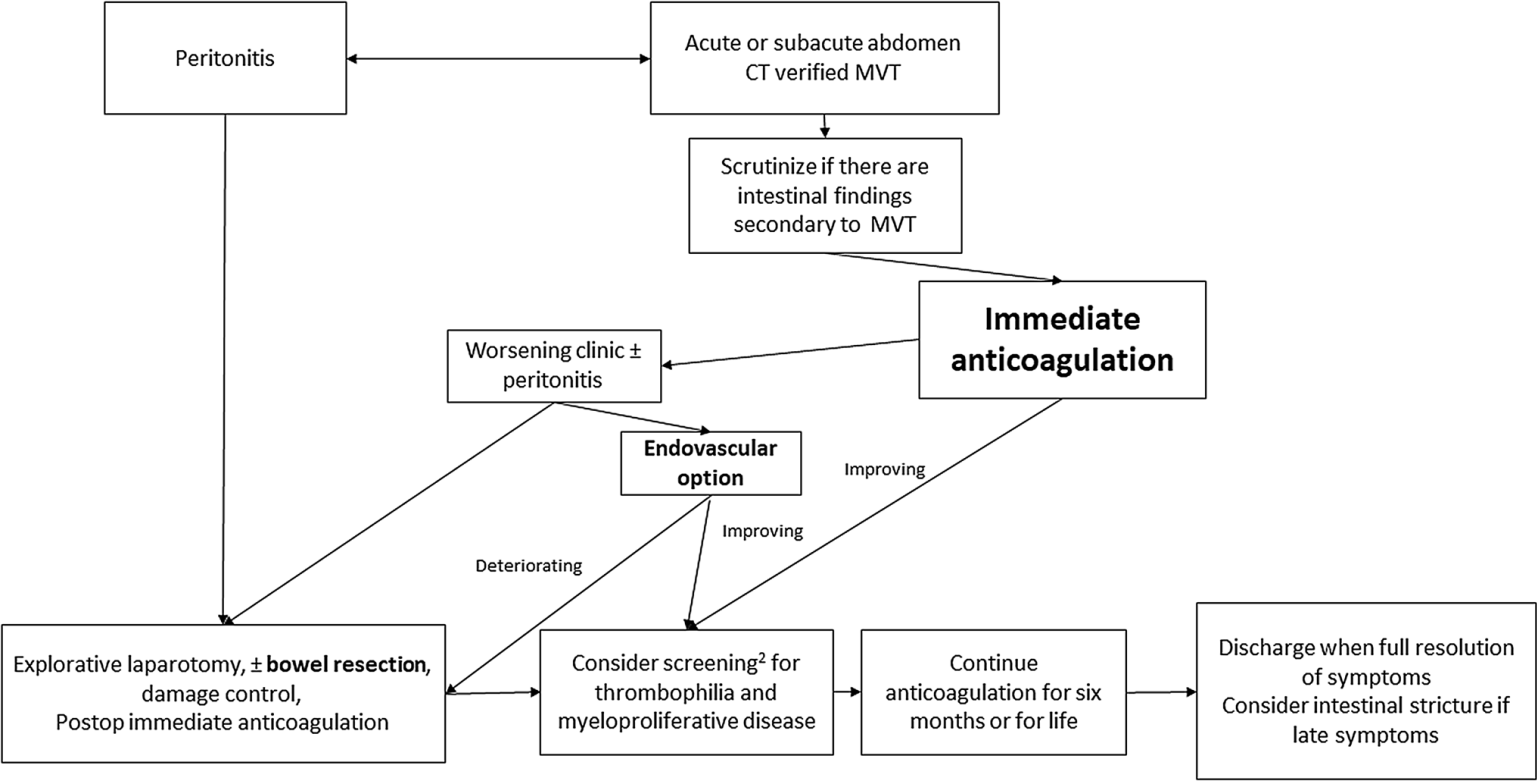
Proposed management algorithm in patients with acute MVT

Endovascular therapy was selectively performed in a few patients and proved to be successful in the majority of these, in whom bowel resection could be avoided. Two patients were operated after 3 and 5 months, respectively, due to late development of severe intestinal stricture with ileus (Fig. 3). CT features such as extensive thrombosis and ascites seem to be predictive factors of poor recanalization on anticoagulant therapy [20]. Clinicians should be aware of the severity of thrombotic and intestinal ischemic lesions on the CT images to be able to proceed with more aggressive approaches, either with endovascular therapy or laparotomy with bowel resection when needed. The 30-day mortality of 3.2% in the present study supports a conservative anticoagulation-first treatment approach (Fig. 4). Endovascular therapy may have a role in patients with extensive portomesenteric thrombosis at diagnosis, but this has to be proven in a large multicenter randomized trial.

The limitations of the present study include mainly its retrospective design. Information on bleeding complications due to anticoagulation therapy was not possible to accurately retrieve. The sample sizes of the patients in the two periods were probably not sufficiently large to be able to show a difference in bowel resection rates. Assuming that the six patients primarily diagnosed at autopsy in the former period would have undergone bowel resection if timely diagnosed, the bowel resection rate would have been significantly higher in the former compared to the latter period (20/58 vs. 10/62, respectively, *p *=  0.02). The low autopsy frequency during the latter time period might have led to an underestimation of the contemporary 30-day mortality in comparison with the former time period when autopsies were more frequently conducted. Nevertheless, the comparably large sample size in our study enabled us to evaluate our study results with multivariable testing.

In conclusion, short-term prognosis in patients with MVT seems to have improved. Contemporary data show that immediate anticoagulation is an effective first-line therapy in patients with MVT.
